# Supplementary material for: Metabolic cost calculations of gait using musculoskeletal energy models, a comparison study
Source: PLoS One. 2019 Sep 18;14(9):e0222037. doi: 10.1371/journal.pone.0222037 (PMC6750598; doi:10.1371/journal.pone.0222037)
Supplement: S1 File — A description of the metabolic energy models that were compared. (PDF) [file pone.0222037.s001.pdf]

S1: Metabolic Energy Models

S1.1: Model BHAR04

Model BHAR04 calculates the metabolic rate in W as a sum of the activation heat rate, $\dot{h}_A$, the maintenance heat rate, $\dot{h}_M$, the shortening/lengthening heat rate, $\dot{h}_{SL}$, and the work, w [1].

$$\dot{E} = \dot{h}_A + \dot{h}_M + \dot{h}_{SL} + w \tag{1}$$

The activation heat rate is determined as follows:

$$\dot{h}_A = \phi m_{mus}(A_f u_f f_{FT} + A_s u_s f_{ST}) \tag{2}$$

where ϕ is a decay function, m_{mus} is the muscle mass, $A_f = 133$ W/kg and $A_s = 40$ W/kg are activation heat rate constant for fast twitch and slow twitch muscles, respectively, u_f and u_s are the stimulation levels of the fast twitch and slow twitch muscles, and f_{FT} and f_{ST} are the percentages of fast twitch and slow twitch muscles [1].

ϕ is determined as follows:

$$\phi = 0.06 + \exp\left(\frac{-t_{stim}u}{\tau_\phi}\right) \tag{3}$$

where t_{stim} is the time the muscle is activated above 0.1, $u(t)$ is the muscle stimulation, and τ_ϕ is the decay time constant [1].

The stimulation of the slow twitch and fast twitch muscles are determined as follows:

$$u_f = 1 - \cos\left(\frac{\pi}{2}u(t)\right) \tag{4}$$

$$u_s = \sin\left(\frac{\pi}{2}u(t)\right) \tag{5}$$

The maintenance heat rate is determined as follows:

$$\dot{h}_M = l_M m_{mus}(M_f u_f f_{FT} + M_s u_s f_{ST}) \tag{6}$$

where l_M is the length dependence of the maintenance heat rate (see Fig. 2 in [1]), and $M_f = 111$ W/kg and $M_s = 74$ W/kg are maintenance heat rate constants [1].

The shortening-lengthening heat rate is determined as follows:

$$\dot{h}_{SL} = -\alpha v_{CE} \tag{7}$$

where v_{CE} is the contractile element velocity, negative for shortening, and α is a constant that is different for shortening and lengthening:

$$\alpha = 0.16a(t)F_{max}f(l_{CE}(t)) + 0.18F_{CE}(t) \quad v_{CE} \leq 0 \tag{8}$$

$$\alpha = 0.157F_{CE} \quad v_{CE} > 0 \tag{9}$$

where $a(t)$ is the muscle activation, F_{max} is the maximum isometric force, $f(l_{CE}(t))$ is the force-length relationship, and $F_{CE}(t)$ the current force in the muscle [1].

Finally, the work rate is determined as follows:

$$w = -v_{CE}F_{CE} \tag{10}$$

S1.2: Model HOUD06

Model HOUD06 [2] calculates the metabolic rate in W as a sum of the activation heat rate, $\dot{h}_A$, the maintenance heat rate, $\dot{h}_M$, the shortening/lengthening heat rate, $\dot{h}_{SL}$, and the work, w.

$$\dot{E} = \dot{h}_A + \dot{h}_M + \dot{h}_{SL} + w \tag{11}$$

Table S1.1. Constants for short twitch and fast twitch fibers.

Variable	Fast twitch	Short twitch
$\bar{h}_A$	52.5 W/kg	10.98 W/kg
$\bar{h}_M$	97.5 W/kg	13.42 W/kg
k_1	12	6
k_2	14	8
$\bar{h}_{SL}$	0.28 F_{max}	0.16 F_{max}

The activation heat rate is determined as follows:

$$\dot{h}_A = m_{mus}\bar{h}_A\nu\frac{1-\exp(-0.25-\frac{18.2}{\nu\nu_{max}})}{1-\exp(-0.25-\frac{18.2}{\nu_{max}})} \tag{12}$$

where $\bar{h}_A$ is the activation heat rate constant, $\nu = a(t)^2$ is the relative stimulation frequency, and $\nu_{max} = k_1 + k_2 a(t)$ is the maximum stimulation frequency. These constants are defined for short twitch and fast twitch fibers and calculated for each muscles by taking the product of the constant for short twitch fibers and the percentage of short twitch fibers, and adding to this the product of the constant for fast twitch fibers and the percentage of fast twitch fibers. These values are given in table S1.1 [2].

The maintenance heat rate is determined as follows:

$$\dot{h}_M = m_{mus}(\bar{h}_A + \bar{h}_M)a(t)\left(f(l_{CE}(t)) - \frac{\bar{h}_A}{\bar{h}_A + \bar{h}_M}\right) \tag{13}$$

where $\bar{h}_M$ is the maintenance heat rate constant, different for short twitch and fast twitch fibers, as given in table S1.1 [2].

The shortening-lengthening heat rate is calculated as follows:

$$\dot{h}_{SL} = \bar{h}_{SL}a(t)f(l_{CE}(t)) - v_{CE} \qquad \text{where: } v_{CE} < 0 \tag{14}$$

where $\bar{h}_{SL}$ is given in table S1.1 for short twitch and fast twitch fibers [2].

S1.3: Model UMBE03

Model UMBE03 determines the metabolic rate per muscle in W as follows:

if $l_{CE} \leq l_{CE(OPT)}$

$$\dot{E} = m_{mus}(h_{AM}A_{AM}S + h_{SL}S) - w \tag{15}$$

if $l_{CE} > l_{CE(OPT)}$

$$\dot{E} = m_{mus}((0.4h_{AM} + 0.6h_{AM}f(l_{CE}))A_{AM}S + h_{SL}Sf(l_{CE})) - w$$

where $\dot{h}_{AM}$ is the activation-maintenance heat rate, $A_{AM} = A^{0.6}$, and S is a scaling factor, equal to 1.5 in aerobic conditions. When the fiber length is longer than optimal, $\dot{h}_{AM}$ is split up into two parts, 40% represents the activation heat rate, while 60% represents the activation heat rate, which is dependent on the location on the force-length relationship [3].

The following equation is used to find the activation-maintenance heat rate [3]:

$$\dot{h}_{AM} = 128f_{FT} + 25 \tag{16}$$

The shortening-lengthening heat rate, $\dot{h}_{SL}$ is different for shortening and lengthening velocity and is determined as follows:

$$\dot{h}_{SL} = \begin{cases} \left(-\alpha_{S(ST)}\tilde{v}_{CE}(1-f_{FT}) - \alpha_{S(FT)}\tilde{v}_{CE}f_{FT}\right)A_s & \text{if } \tilde{v}_{CE} \leq 0 \\ \alpha_L\tilde{v}_{CE}A & \text{if } \tilde{v}_{CE} > 0 \end{cases} \tag{17}$$

where the first term, $\alpha_{S(ST)}\tilde{v}_{CE}(1 - f_{FT})$, cannot exceed 100 W/kg [3].

$\tilde{v}_{CE} = v_{CE}/l_{CE(OPT)}$ is the muscle fiber velocity in s^{-1} normalized to the optimal fiber length, $l_{CE(OPT)}$. $\alpha_{S(ST)} = 100/\tilde{v}_{CE(MAX-ST)}$ and $\alpha_{S(FT)} = 153/\tilde{v}_{CE(MAX-FT)}$ are the shortening heat coefficients for slow twitch (ST) and FT fibers, respectively, in J/kg. They are dependent on the maximum fiber velocity of ST and FT fibers. $\alpha_L = 4\alpha_{S(ST)}$ is the lengthening heat coefficient, which is based on experimental data [3].

A and A_S are scaling factors that depend on the stimulation and activation:

$$A = \begin{cases} u & \text{when } u > a \\ (u+a)/2 & \text{when } u \leq a \end{cases} \tag{18}$$

$$A_S = \begin{cases} A^2 & \text{if } \tilde{v}_{CE} \leq 0 \\ A & \text{if } \tilde{v}_{CE} > 0 \end{cases} \tag{19}$$

S1.4: Model LICH05

Shortening velocity is positive in model LICH05 [4]. This model calculates the metabolic rate as a sum of the heat rate, $\dot{h}$, normalized to optimal fiber length and maximum isometric force, and the work:

$$\dot{E} = \dot{h} l_{CE(OPT)} F_{max} + w \tag{20}$$

The heat rate is a sum of the shortening-lengthening heat rate and the maintenance heat rate:

$$\dot{h} = (0.3a\dot{h}_M + 0.7af(l_{CE}(t))\dot{h}_M + af(l_{CE}(t))\dot{h}_{SL} \tag{21}$$

where 30% of the maintenance heat rate represents activation, which is only scaled by the activation [4].

The maintenance heat rate is determined as follows:

$$\dot{h}_M = \begin{cases} \gamma\frac{\tilde{v}_{CE(max)}}{G^2} & \text{if:} \quad v_{CE}(t) > 0 \\ 0.3\gamma\frac{\tilde{v}_{CE(max)}}{G^2} + 0.7\gamma\frac{\tilde{v}_{CE(max)}}{G^2}\exp(-7\tilde{v}_{CE(max)}(g(v_{CE}(t)) - 1)) & \text{if:} \quad v_{CE}(t) \leq 0 \end{cases} \tag{22}$$

where γ is a heat rate value that decays with the stimulation time, $G = 4$ is the curvature of the force-velocity curve, and $g(v_{CE})$ is the location on the force-velocity relationship [4].

γ is determined as follows [4]:

$$\gamma = 0.8\exp(-0.72t_{stim}) + 0.175\exp(-0.022t_{stim}) \tag{23}$$

The shortening-lengthening heat rate is determined as follows [4]:

$$\dot{h}_{SL} = \begin{cases} \frac{\tilde{v}_{CE}}{G} & \text{if:} \quad v_{CE}(t) > 0 \\ -0.5g(v_{CE}(t))v_{CE}(t) & \text{if:} \quad v_{CE}(t) \leq 0 \end{cases} \tag{24}$$

References

1. Bhargava LJ, Pandy MG, Anderson FC. A phenomenological model for estimating metabolic energy consumption in muscle contraction. Journal of Biomechanics. 2004;37(1):81–88.

2. Houdijk H, Bobbert M, De Haan A. Evaluation of a Hill based muscle model for the energy cost and efficiency of muscular contraction. Journal of Biomechanics. 2006;39(3):536–543.

3. Umberger BR, Gerritsen KG, Martin PE. A model of human muscle energy expenditure. Computer methods in biomechanics and biomedical engineering. 2003;6(2):99–111.

4. Lichtwark GA, Wilson A. A modified Hill muscle model that predicts muscle power output and efficiency during sinusoidal length changes. Journal of Experimental Biology. 2005;208(15):2831–2843.
