# Supplementary material for: Metabolic cost calculations of gait using musculoskeletal energy models, a comparison study
Source: PLoS One. 2019 Sep 18;14(9):e0222037. doi: 10.1371/journal.pone.0222037 (PMC6750598; doi:10.1371/journal.pone.0222037)
Supplement: S2 File — A description of the muscle model that was used to determine the stimulation, activation, and other muscle parameters. (PDF) [file pone.0222037.s002.pdf]

S2: Muscle Model

Fig. S2.1 shows the three element Hill-type muscle that was used in this work. It consists of a contractile element, parallel elastic element and series elastic element. Its input is the stimulation, u, and the states are the activation, a, and the contractile element length, l_{CE}. The force in the contractile element is determined as follows:

$$F_{CE} = af(l_{CE})g(v_{CE})F_{max} \tag{1}$$

where $f(l_{CE})$ is the force-length relationship, and $g(v_{CE})$ is the force-velocity relationship.

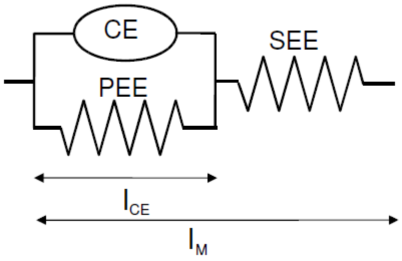

Fig S2.1. Three element Hill-type muscle with contractile element, parallel elastic element, and series elastic element.

The force-length relationship was as follows:

$$f(l_{CE}) = \exp\left(\frac{-(l_{CE} - l_{CE(OPT)})^2}{(Wl_{CE(OPT)})^2}\right) \tag{2}$$

The force-velocity equation was defined as follows:

$$g(v_{CE}) = \begin{cases} \frac{v_{CE(max)}+v_{CE}}{v_{CE(max)}-v_{CE}/A_{hill}} & \text{if} \quad v_{CE} < 0 \\ \frac{g_{max}v_{CE}+c}{v_{CE}+c} & \text{if} \quad v_{CE} \geq 0 \end{cases} \tag{3}$$

$$\text{with} \quad c = \frac{v_{CE(max)}A_{hill}(g_{max}-1)}{A_{hill}+1} \tag{4}$$

The parallel and series elastic elements are modeled as quadratic springs. Their force are found based on the model presented by McLean et al. [1]:

$$F(l) = \begin{cases} k_1(l - l_{slack}) & if l \leq l_{slack} \\ k_1(l - l_{slack}) + k_2(l - l_{slack})^2 & if l > l_{slack} \end{cases} \tag{5}$$

where l denotes the length of the element and l_{slack} the slack length. k_1 and k_2 are stiffness constants. $k_1 = 0.01$ F_{max}/m represents a small linear stiffness, which was added to aid the optimization. It is equal to. k_2 is equal to the following:

$$k_2(PEE) = \frac{F_{max}k_{PEE}}{l^2_{CE(OPT)}} \tag{6}$$

$$k_2(SEE) = \frac{F_{max}}{(u_{max}l_{CE(OPT)})^2} \tag{7}$$

where $k_{PEE} = 1$ and $u_{max} = 0.04$ are dimensionless constants.

The muscle mass, m_{mus} was determined using the maximum isometric force and the optimal fiber length:

$$m_{mus} = \frac{F_{max}}{\sigma}\rho l_{CE(OPT)} \tag{8}$$

where $\sigma = 25$ N/cm^2 is the muscle-specific stress [2] and $\rho = 1059.7$ kg/m^3 is the density of muscle.

Tab. S2.1 shows the maximum isometric force F_{max}, optimal fiber length, $l_{CE(OPT)}$, width of the force-length curve, the slack length of the parallel elastic element (PEE) and the series elastic element (SEE), the nominal muscle length, l_m, and the percentage of fast twitch fibers for each muscle. Several parameters parameters for each muscle. Several parameters were the same for each muscle, the activation time, $T_{act} = 0.01$ s, the deactivation time, $T_{deact} = 0.03$ s, the maximum shortening velocity, $v_{CE(max)} = 12\ l_{CE(OPT)}$/s, the maximum force during lengthening, $g_{max} = 1.5$ F_{max}, and the normalized hill constant, $A_{hill} = 0.25$.

Table S2.1. Muscle Parameters

Muscle	$F_{max}[N]$	$l_{CE(OPT)}$ [m]	Width	PEE slack [$l_{CE(OPT)}$]	SEE slack [m]	l_m [m]	% FT fibers
Iliopsoas	1500	0.102	1.298	1.2	0.142	0.248	0.5
Gluteals	3000	0.2	0.625	1.2	0.157	0.271	0.45
Hamstrings	3000	0.104	1.197	1.2	0.334	0.383	0.35
Rectus Femoris	1200	0.081	1.443	1.4	0.398	0.474	0.65
Vastus	7000	0.093	0.627	1.4	0.223	0.271	0.5
Gastrocnemius	3000	0.055	1.039	1.2	0.42	0.487	0.5
Soleus	4000	0.055	1.039	1.2	0.245	0.284	0.2
Tibialis Anterior	2500	0.082	0.442	1.2	0.317	0.381	0.25

References

1. McLean S, Su A, van den Bogert A. Development and validation of a 3-D model to predict knee joint loading during dynamic movement. Journal of Biomechanical Engineering. 2003;125(6):864–874.

2. Nigg BM, Herzog W. Biomechanics of the musculo-skeletal system. John Wiley & Sons; 2007.
