# Supplementary figures and images for: Metabolic cost calculations of gait using musculoskeletal energy models, a comparison study
Source: PLoS One. 2019 Sep 18;14(9):e0222037. doi: 10.1371/journal.pone.0222037 (PMC6750598; doi:10.1371/journal.pone.0222037)

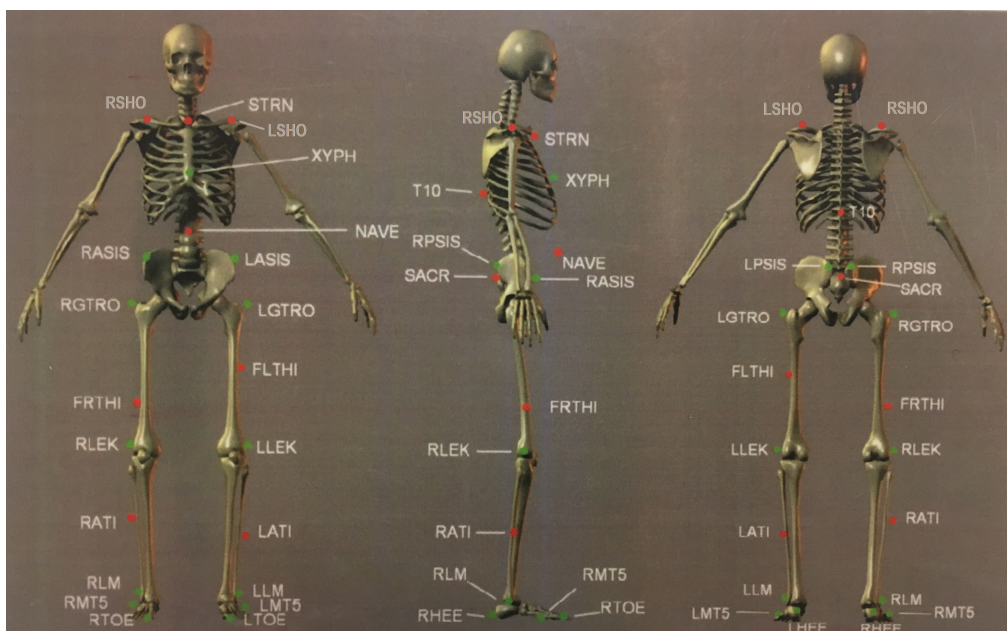

**Fig S1: Placement of markers on the body**

Supplement: S1 Fig — Illustration of the placement of the markers that were used in the experiment on the body. (PDF) [file pone.0222037.s004.pdf]
